# Case Report: A case of transcatheter aortic valve replacement in a patient with a small aortic annulus using the Acurate Neo2

**DOI:** 10.3389/fcvm.2025.1513802

**Published:** 2025-02-06

**Authors:** Shahzil Abdur Rehman Malik, Jamshed Ali, Maleeha Javed, Kamran Hussain, Navaira Azeem, Nasir Rehman, Osman Faheem

**Affiliations:** ^1^ Medical College, Aga Khan University, Karachi, Pakistan; ^2^ Section of Cardiology, Department of Medicine, Aga Khan University, Karachi, Pakistan

**Keywords:** self-expanding transcatheter heart valves, prosthesis-patient mismatch, small aortic annulus, resource limited settings, case report

## Abstract

Small aortic annulus poses a major challenge in aortic valve replacement due to the increased risk of prosthesis–patient mismatch (PPM) and increased surgical risk. In recent years, transcatheter aortic valve replacement (TAVR) has emerged as a popular alternative to the traditional surgical aortic valve replacement. We present the case of an 80-year-old woman with a small aortic annulus who underwent TAVR using a self-expanding transcatheter heart valve Acurate Neo2 after an non-ST-segment elevation myocardial infarction (NSTEMI) presentation. These risks, combined with advanced age, significant co-morbidities, and a severely calcified small aortic annulus, supported the choice of TAVR with a self-expanding Acurate Neo2 valve. Despite multiple risk factors for PPM, the patient had a successful outcome without major complications. Our case highlights the off-label use of the Acurate Neo2 valve in one of the smallest aortic annuli reported to date, showcasing its feasibility in Asian and resource-limited settings.

## Introduction

Small aortic annulus (SAA) is defined as an aortic annulus unable to accommodate a prosthesis >21 mm or an annulus diameter ≤23 mm. SAA is related to an increased surgical risk and a greater chance of suboptimal valve hemodynamics, including a high incidence of moderate-to-severe prosthesis–patient mismatch (PPM) (1). Greater severity of PPM translates into an increased perioperative and overall mortality rate. Various predictors of PPM include older age, female sex, hypertension, diabetes, renal failure, larger body surface area, larger body mass index, and the utilization of a bioprosthetic valve (2). Treatment options historically included a surgical aortic valve replacement (SAVR). However, more recently, transcatheter aortic valve replacement (TAVR) has appeared as an alternative approach, producing more favorable hemodynamic results in patients with SAA, compared with those of SAVR (1).

Self-expanding supra- and intra-annular transcatheter heart valves (THV) have demonstrated better hemodynamics in patients with SAA (3, 4). We present the case of a patient with SAA and multiple risk factors for PPM, who underwent TAVR 2 weeks after presenting to the emergency room with a non-ST-segment elevation myocardial infarction (NSTEMI). The procedure was performed using a self-expanding (SE) THV Accurate Neo2 in a thickened, calcified, and severely stenotic aortic valve.

## Case description

We present the case of an 80-year-old woman who presented to the emergency room (ER) with complaints of chest pain for 30 min, localized to the center of the chest. The pain was described as dull and aching in nature and recurred usually after heavy meals. The patient also had symptoms of paroxysmal nocturnal dyspnea (PND) for the past 2 days. She was class II according to the New York Health Association Functional Classification and had diabetes mellitus and hypertension. She had a history of breast cancer treated with modified radical mastectomy and radiation therapy and a cardioembolic stroke the previous year.

On arrival at the ER, the patient was hemodynamically stable and had no significant physical examination findings. ECG showed ST depressions in V5-V6, and troponin I was markedly elevated. Therefore, the patient was managed on the lines of NSTEMI. She was admitted to the Cardiac Care Unit and kept under observation.

**Figure 1:**
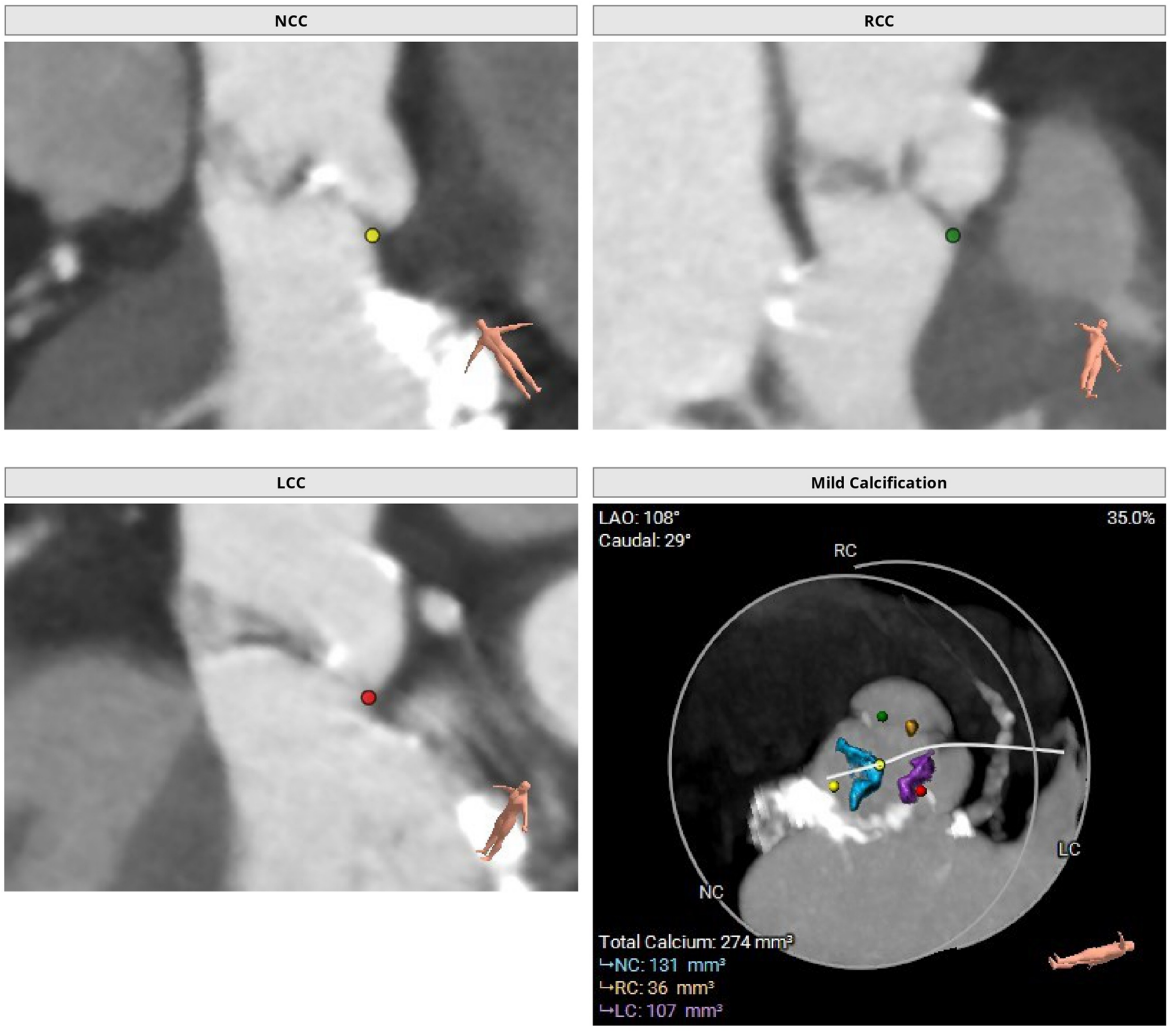
Preoperative thoraco-abdominal CT images were analyzed using 3mensio Structural Heart software (Pie Medical Imaging, the Netherlands). Preoperative thoraco-abdominal CT images showed a tricuspid aortic valve with mild valvular calcifications.

**Figure 2:**
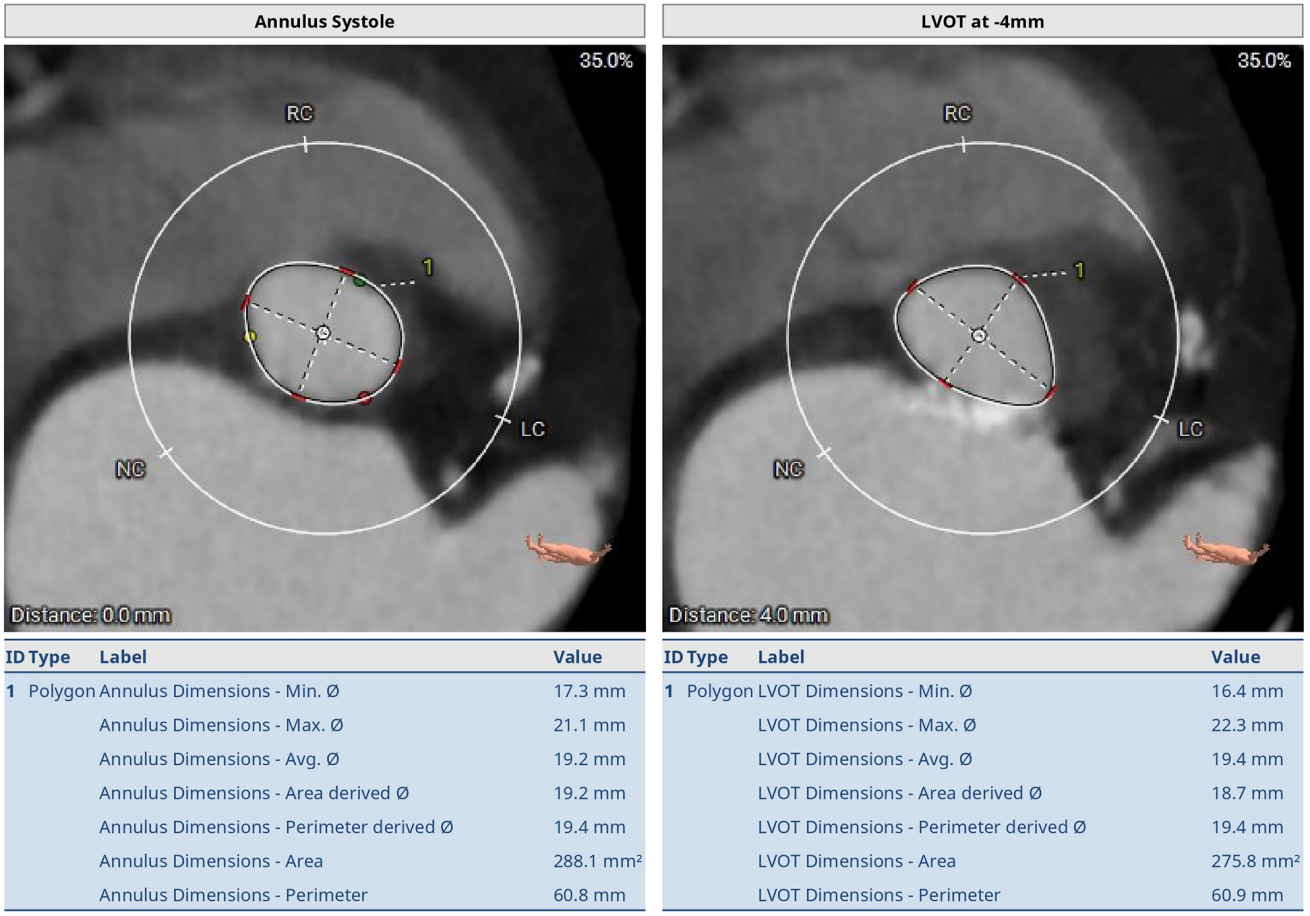
Preoperative thoraco-abdominal CT images showing the annular measurements at systole. The aortic annulus perimeter was 60.8 mm (minor annulus diameter 17.3 mm, major annulus diameter 21.1 mm) and the area was 288.1 mm^2^. The LVOT perimeter was 60.9 mm (minor annulus diameter 16.4 mm, major annulus diameter 22.3 mm) and the area was 275.8 mm^2^.

**Figure 3:**
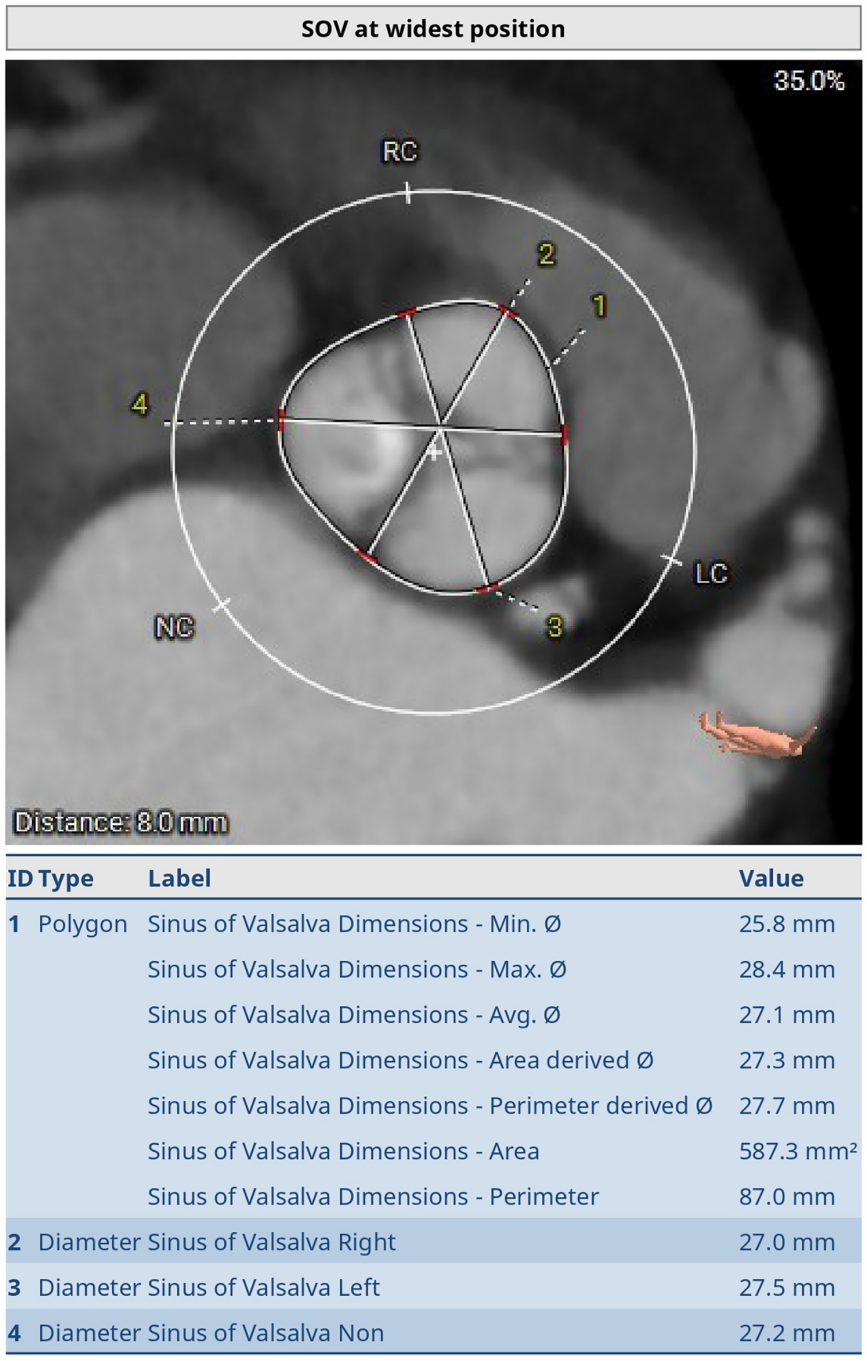
Preoperative thoraco-abdominal CT image showing the measurements of sinus of Valsalva (SoV) at the widest position. The SoV perimeter was 87.0 mm (minor SoV diameter 25.8 mm, major SoV diameter 21.1 mm) and the area was 587.3 mm^2^. Diameters of the right SoV, left SoV, and non-coronary SoV were 27.0 mm, 27.5 mm, and 27.2 mm respectively.

Transthoracic echocardiography was carried out, which showed a thickened, calcified, and severely stenotic aortic valve, measuring 0.7 cm^2^ (normal range 2.5–4.5 cm^2^), and peak and mean pressure gradients of 55 and 40 mmHg (normal range <5 mmHg). The Vmax of the aortic valve was 3.8 m/s (normal range <2 m/s). Preoperative thoraco-abdominal computed tomography angiography (CTA) showed an aortic annulus perimeter of 55.4 mm, ventricular outflow tract of 13.5 mm, short-axis diameter of 15.1 mm, and long-axis diameter of 20.2 mm at the annulus. Sinus width was 25 mm, diameter of the sinotubular junction was 17.8 mm, the ascending aorta was 24 mm wide, and sinus height was 14.3 mm. The distance to the first coronary (right) was 16.1 mm. The aortic annulus perimeter was 60.8 mm (minor annulus diameter 17.3 mm, major annulus diameter 21.1 mm) and the area was 288.1 mm^2^. The LVOT perimeter was 60.9 mm (minor annulus diameter 16.4 mm, major annulus diameter 22.3 mm) and the derived area was 275.8 mm^2^ (Figures 1–3).

Left heart catheterization was carried out, which showed distal left main and osteoproximal left anterior descending disease. Intravascular ultrasound (IVUS) showed that it was non-flow limiting and medical therapy was planned. Carotid Doppler showed no significant plaque or high-grade stenoses and normal CCA and ICA indices.

The patient's preoperative evaluation included a calculated Society of Thoracic Surgery (STS) risk score of 5.4% for operative mortality and 12.7% for combined morbidity and mortality risk, categorizing her as intermediate surgical risk. Given the STS score, frailty, and small annulus, it was decided to proceed with a TAVR using a SE small-sized 23 mm Acurate Neo2 (Boston Scientific, Marlborough, MA, USA) valve.

**Figure 4:**
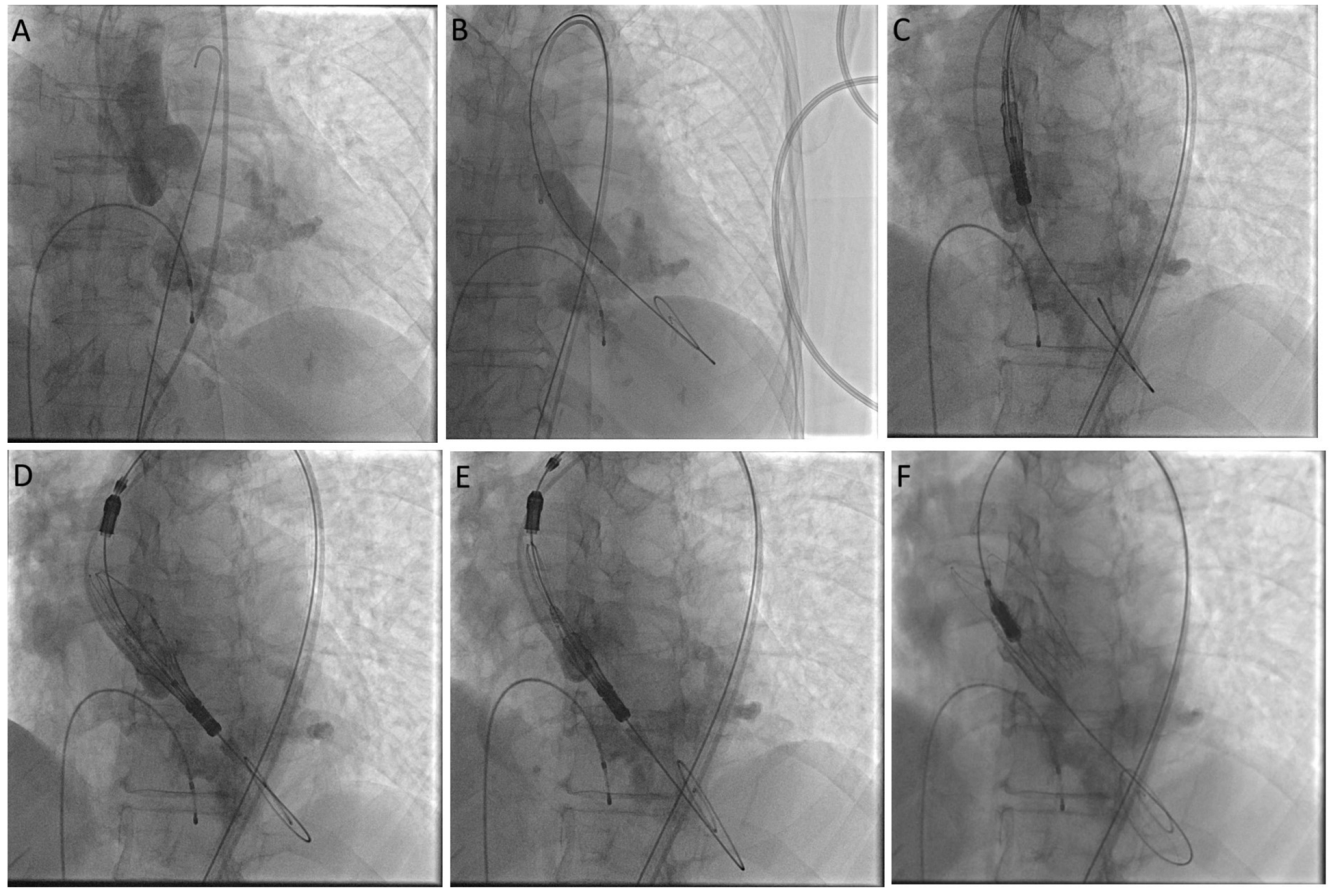
Cine images showing aortogram in cusp overlap view **(A)**, steps of balloon valvuloplasty **(B)**, and valve deployment **(C–F)**.

After inducing general anesthesia, ultrasound-guided access to the left common femoral arterial (CFA) was obtained. Right CFA and femoral venous accesses were also obtained. Left CFA access was preclosed using two ProGlides. A 14 Fr sheath was inserted in the left CFA access. A 5 Fr pigtail catheter was inserted for hemodynamic monitoring. The valve was crossed with a JR4 Catheter and a straight tip 0.035 inch wire. The pre-procedure LV-Ao gradient was 40 mmHg. The JR catheter was exchanged to a pigtail 6 Fr catheter, and a Safari 0.038 inch guidewire was inserted over the pigtail catheter. Working projection was LAO 31° CRA 8°. Under rapid pacing, a balloon aortic valvuloplasty (BAV) was carried out using a VACS 16×40 mm balloon. A self-expanding THV (Acurate Neo2, 23 mm, Boston Scientific) was implanted in the supra-annular location of the leaflets. The valve was implanted using the commissural alignment technique (Figure 4). No immediate complications were seen. The LV-Ao gradient was 2 mmHg. No aortic insufficiency was noted. A lower aortogram was conducted, which showed a left iliac artery dissection and a contained perforation that was managed using local intermittent tamponade with balloon inflations.

Since discharge, the patient has remained clinically stable and has reported marked improvements in her previous symptoms of shortness of breath and chest pain. A 2-month postprocedure transthoracic echocardiography showed normal left ventricular systolic function (ejection fraction 55%–60%) and a normally function bioprosthetic Acurate Neo2 valve at the aortic position with a circular orifice. Hemodynamic assessment revealed a peak pressure gradient of 12 mmHg and a mean pressure gradient of 7 mmHg, Vmax 2.4 m/s, and the Dimensionless Velocity Index (DVI) was 0.54 indicating excellent valve function. No aortic regurgitation or paravalvular leakage was observed.

## Discussion

It has been observed that Asian populations have a smaller aortic annulus compared to Western populations. This, coupled with differences in physique, the anatomy of the aortic complex, thrombogenicity, and susceptibility to bleeding poses additional risks for Asian populations undergoing aortic valve replacement. Compared to their Western counterparts, Asian populations also have higher rates of conventional risk factors at a younger age, such as congestive heart failure, chronic kidney disease, smoking, hypertension, obesity, and diabetes mellitus (5). Given these challenges, it is extremely important to establish a safe and reliable strategy to deliver optimal treatment to Asian populations undergoing aortic valve replacement. Compared to SAVR, patients who underwent TAVR have shown comparable results, including a reduction in mortality in patients with severe aortic stenosis. TAVR is preferred for patients who may not be surgical candidates or be at elevated risk due to advanced age and co-morbid conditions such as our patient. In addition, lower in-hospital mortality rates, fewer complications, and a shorter in-hospital stay also favor TAVR over SAVR (6, 7).

The STS risk of 5.4% for operative mortality and 12.7% for morbidity and mortality in our patient aligns with the intermediate risk profile often observed in elderly patients with severe co-morbidities undergoing cardiac surgery. Severe frailty was another strong factor that made our patient a poor surgical candidate. This decision was further supported by the presence of a small and severely calcified aortic annulus, which poses a unique challenge for valve replacement and lifetime management of severe AS (8).

In resource-limited settings such as a low- and middle-income countries like Pakistan, balloon-expandable valves are often unavailable. Furthermore, moderate and severe PPM have been shown to have a lower incidence with the use of self-expanding compared with balloon-expandable valves, despite having higher rates of paravalvular leaks (9). The Acurate Neo2 has already been widely compared to existing balloon-expandable and self-expanding valves and has shown comparable outcomes (10). The supra-annular location of the self-expanding Acurate Neo2 provides an advantage over the balloon expandable valves and intra-annular self-expanding valves, allowing optimal expansion and ensuring a larger effective orifice area, which is particularly beneficial in preventing PPM in patients with SAA (11). The Acurate Neo2 prosthesis is typically recommended for use in aortic annuli with a native diameter in the range of 21–27 mm. The annular diameter of our patient was significantly smaller than the recommended company instructions for use (IFU), presenting a unique challenge. Alternative options, such as SAVR or balloon-expandable valves, were either not viable or unavailable in our resource-limited setting, necessitating the use of the Acurate Neo2 despite its off-label application. Our decision to proceed ahead with its off-label use was backed by the study by Eckel et al., in which they demonstrated its low rate of PVL and PPM when implanted in small annuli, even outside the official IFUs. Although the study recognized annular calcification and deep implantation as potential predictors for PPM, we opted to implant the Acurate Neo2 in this patient despite a thickened and heavily calcified annulus, due to the absence of better alternatives (12). The valve's low-profile delivery system, supra-annular design, and ease of future coronary access further strengthened its suitability for this case.

Our case report highlights a successful TAVR in one of the smallest reported aortic annuli. A previous case report reported a successful TAVR in an aortic valve, with an annulus perimeter of 51.7 mm (area of 207.8 mm^2^) and a left ventricular outflow tract (LVOT) perimeter of 45.3 mm with an elliptical shape (area of 149.9 mm^2^). However, the valve lacked any significant calcifications. In comparison, our valve had an aortic annulus perimeter of 55.4 mm, a ventricular outflow tract of 13.5 mm, and had severe calcifications (11).

Common complications associated with TAVR in SAA patients include PPM, valve dislodgement, valve embolization, inadequate stent expansion, and significant paravalvular leakage (PVL), particularly if the aortic valve is highly calcified. PPM results in a greater perioperative and overall mortality rate. Aortic regurgitation and permanent pacemaker implantation (PPMI) may also significantly affect the quality of life of patients and is associated with an overall lower rate of survival (2, 4, 5, 10). In our patient, however, no such complications were observed.

Larger studies have supported the favorable performance and 1-year safety of patients treated with the Acurate Neo2 valve with low rates of 30-day all-cause mortality (3.3% in Mollmann et al. and 0.8% in Kim et al.), 1-year all-cause mortality (11.9% in Mollmann et al. and 5.1% in Kim et al.), and postoperative complications, such as stroke (2.5% in Mollmann et al. and 3.0% in Kim et al.). No patients reported severe paravalvular leak or required re-intervention for valve-related dysfunction and there were no cases of valve thrombosis or endocarditis (13–15).

Guidelines recommend dual antiplatelet therapy (DAPT) consisting of low-dose (75–100 mg once daily) acetylsalicylic acid (ASA) and clopidogrel (75 mg once daily) for the first 3–6 months to protect patients from prosthesis-related thromboembolic events, followed by lifelong single antiplatelet therapy (SAPT) in patients without an underlying indication of chronic oral anticoagulation (OAC) (16). Keeping in line with these guidelines, we also initiated DAPT with ASA and clopidogrel postoperatively in our patient.

Our case report highlights the critical requirement of a safe and reliable strategy to provide optimal treatment to Asian populations undergoing TAVR, particularly in resource-limited settings, where access to certain types of valves may be limited. Despite facing pre-existing challenges, such as small aortic annuli and more conventional risk factors for aortic stenosis, Asian populations may benefit from the use of the Acurate Neo2. While complications may occur, especially in a patient with SAA, individualized assessment and tailored interventions can lead to successful outcomes as shown in our patient.

## Data Availability

The original contributions presented in the study are included in the article, further inquiries can be directed to the corresponding author.

## References

[1] Freitas-Ferraz AB Tirado-Conte G Dagenais F Ruel M Al-Atassi T Dumont E Aortic stenosis and small aortic annulus. Circulation. (2019) 139(23):2685–702. 10.1161/CIRCULATIONAHA.118.038408 31157994

[2] Dayan V Vignolo G Soca G Paganini JJ Brusich D Pibarot P. Predictors and outcomes of prosthesis-patient mismatch after aortic valve replacement. JACC Cardiovasc Imaging. (2016) 9(8):924–33. 10.1016/j.jcmg.2015.10.026 27236530

[3] Voigtländer L Kim WK Mauri V Goßling A Renker M Sugiura A Transcatheter aortic valve implantation in patients with a small aortic annulus: performance of supra-, intra- and infra-annular transcatheter heart valves. Clin Res Cardiol. (2021) 110(12):1957–66. 10.1007/s00392-021-01918-8 34387736 PMC8639544

[4] Regazzoli D Chiarito M Cannata F Pagnesi M Miura M Ziviello F Transcatheter self-expandable valve implantation for aortic stenosis in small aortic annuli: the TAVI-SMALL registry. JACC Cardiovasc Interv. (2020) 13(2):196–206. 10.1016/j.jcin.2019.08.041 31883714

[5] Lee CH Inohara T Hayashida K Park DW. Transcatheter aortic valve replacement in Asia: present status and future perspectives. JACC Asia. (2021) 1(3):279–93. 10.1016/j.jacasi.2021.10.006 36341218 PMC9627874

[6] Warner ED Riley J Liotta M Pritting C Brailovsky Y Jimenez D Aortic valve replacement in patients with ESRD and heart failure with reduced ejection fraction. Am J Cardiol. (2023) 205:111–9. 10.1016/j.amjcard.2023.07.161 37604063

[7] Grigorios T Stefanos D Athanasios M Ioanna K Stylianos A Periklis D Transcatheter versus surgical aortic valve replacement in severe, symptomatic aortic stenosis. J Geriatr Cardiol. (2018) 15(1):76–85. 10.11909/j.issn.1671-5411.2018.01.002 29434629 PMC5803541

[8] Kim H Kang DY Ahn JM Kim JB Yeung AC Nishi T Race-Specific impact of conventional surgical risk score on 1-year mortality after transcatheter aortic valve replacement. JACC: Asia. (2023) 3(3):376–87. 10.1016/j.jacasi.2022.11.007 37323869 PMC10261892

[9] Ferrara J Theron A Porto A Morera P Luporsi P Jaussaud N Prosthesis-patient mismatch in small aortic annuli: self-expandable vs. balloon-expandable transcatheter aortic valve replacement. J Clin Med. (2022) 11(7):1959. 10.3390/jcm11071959 35407567 PMC8999619

[10] Elkoumy A Rück A Abdel-Wahab M Thiele H Rudolph TK Wolf A ACURATE Neo2 transcatheter aortic valve implantation without balloon aortic valvuloplasty—direct ACURATE neo2. Int J Cardiol. (2024) 400:131792. 10.1016/j.ijcard.2024.131792 38244892

[11] Medda M Casilli F Bande M Tespili M Donatelli F. Case report: self-expanding transcatheter valve implantation (Acurate Neo 2) in a very small native aortic annulus. Front Cardiovasc Med. (2023) 10:1195486. 10.3389/fcvm.2023.1195486 37795479 PMC10545879

[12] Eckel C Sötemann D Kim WK Grothusen C Tiyerili V Dohmen G Procedural outcomes of a self-expanding transcatheter heart valve in small annuli. J Clin Med. (2022) 11(18):5313. 10.3390/jcm11185313 36142960 PMC9502952

[13] Möllmann H Holzhey DM Hilker M Toggweiler S Schäfer U Treede H The ACURATE neo2 valve system for transcatheter aortic valve implantation: 30-day and 1-year outcomes. Clin Res Cardiol. (2021) 110(12):1912–20. 10.1007/s00392-021-01882-3 34148125 PMC8639565

[14] Kim WK Möllmann H Montorfano M Ellert-Gregersen J Rudolph TK Van Mieghem NM Outcomes and performance of the ACURATE neo2 transcatheter heart valve in clinical practice: one-year results of the ACURATE neo2 PMCF study. EuroIntervention. (2024) 20(1):85–94. 10.4244/EIJ-D-23-00823 37982152 PMC10756225

[15] Kim WK Tamburino C Möllmann H Montorfano M Ellert-Gregersen J Rudolph TK Clinical outcomes of the ACURATE neo2 transcatheter heart valve: a prospective, multicentre, observational, post-market surveillance study. EuroIntervention. (2023) 19(1):83–92. 10.4244/EIJ-D-22-00914 PMC10173758 36440588

[16] Guedeney P Mehran R Collet JP Claessen BE ten Berg J Dangas GD. Antithrombotic therapy after transcatheter aortic valve replacement. Circ Cardiovasc Interv. (2019) 12(1):e007411. 10.1161/CIRCINTERVENTIONS.118.007411 30630354

